# Supplementary material for: Integrase-associated niche differentiation of endogenous large DNA viruses in crustaceans
Source: Microbiol Spectr. 2023 Dec 8;12(1):e00559-23. doi: 10.1128/spectrum.00559-23 (PMC10871703; doi:10.1128/spectrum.00559-23)
Supplement: Supplemental figures — Figures S1 to S6. [file spectrum.00559-23-s0001.docx]

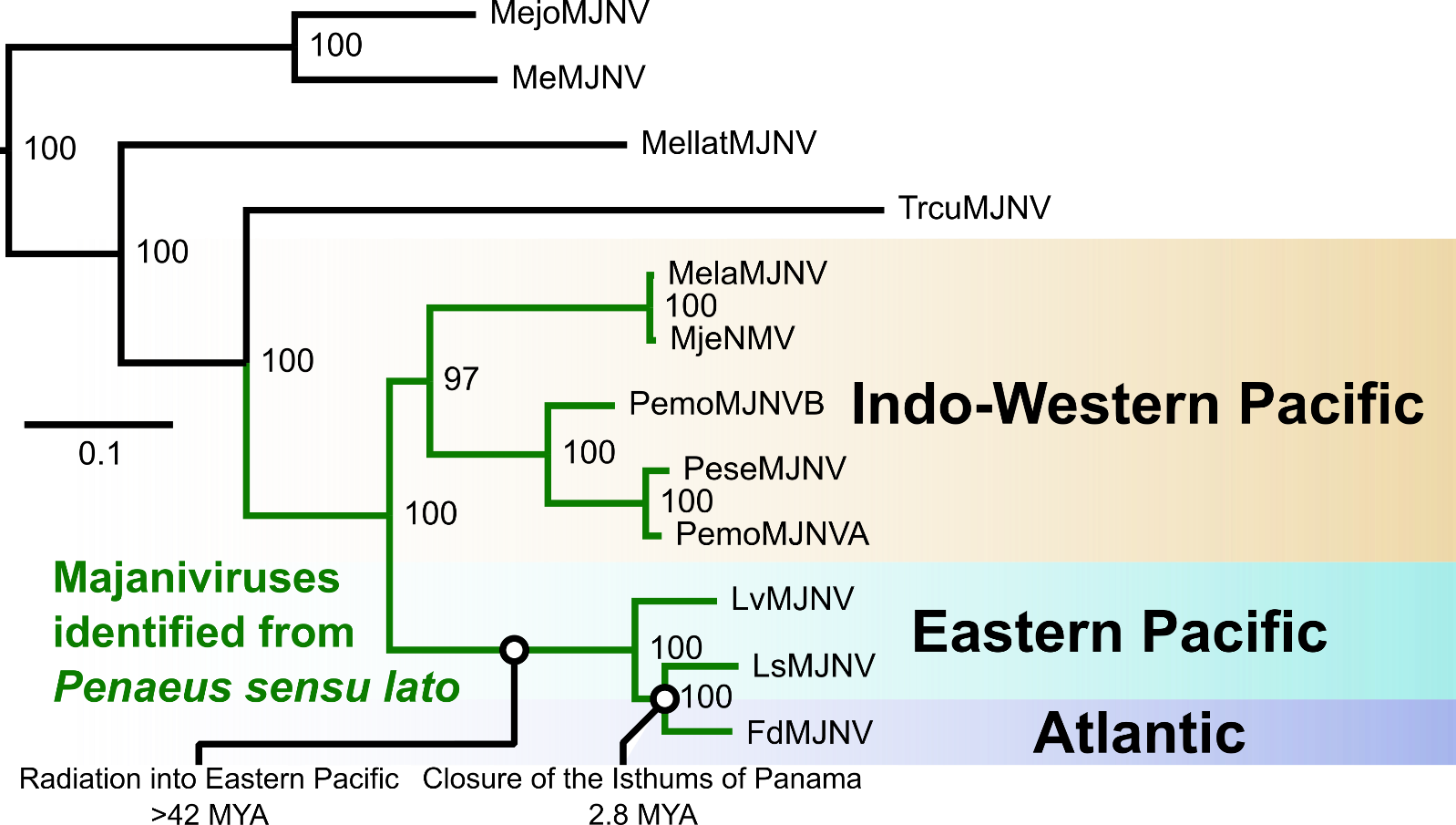


Supplementary Figure 1. Majanivirus phylogeny and host phylogeography

A portion of the maximum-likelihood phylogenetic tree presented in Figure 1 was extracted. Values beside the nodes indicate the ultrafast bootstrap values (1,000 trials).


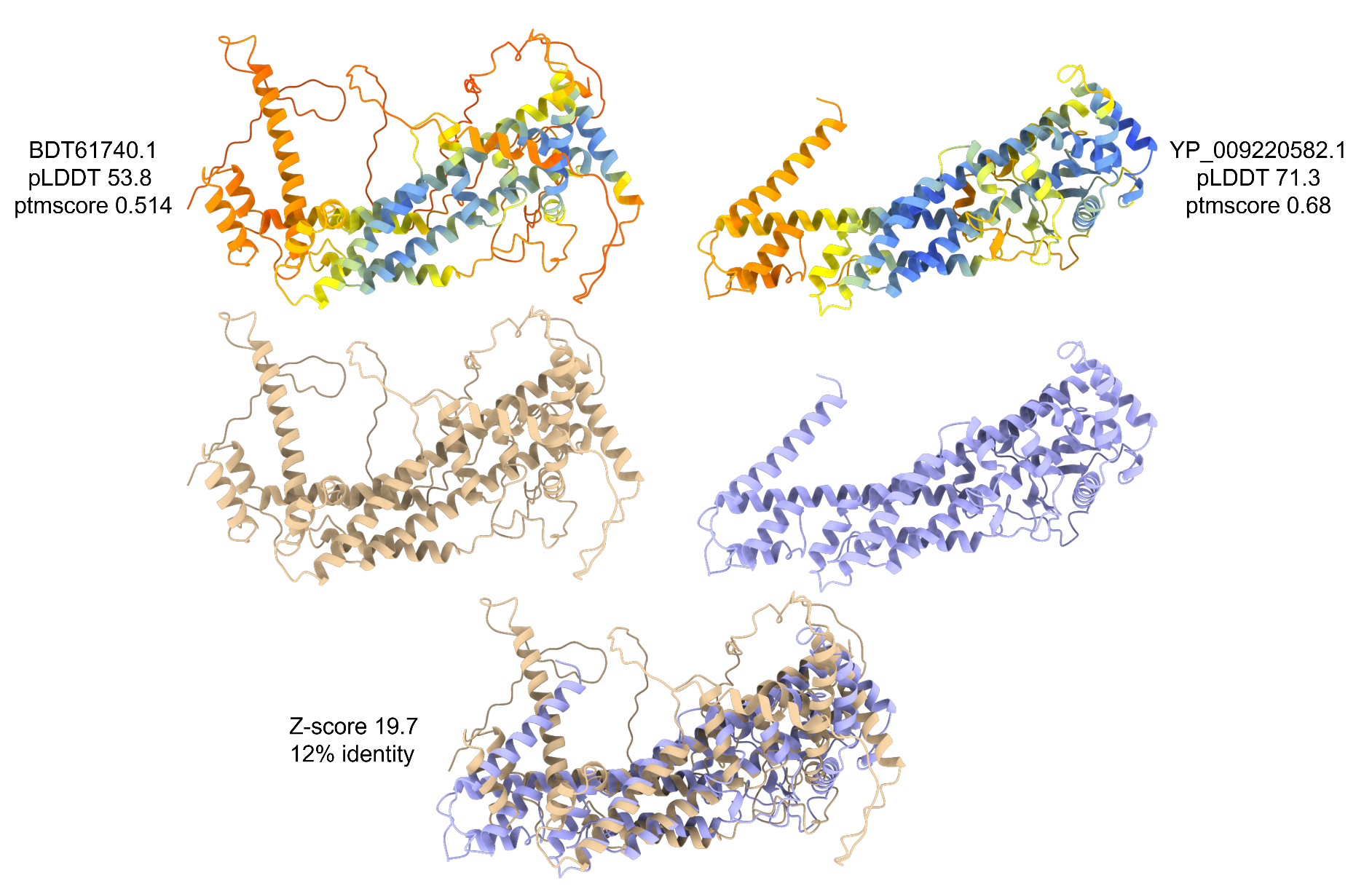


Supplementary Figure 2. Structural predictions and an alignment of wsv308 orthologs.

Top: ColabFold structural predictions of MjeNMV wsv308-like protein (left) and WSSV wsv308 protein (right). Colors indicate the pLDDT confidence measures assigned by ColabFold.

Middle: The same structural predictions as the top, with colors ignored.

Bottom: Matchmaker alignment of the predicted protein structures. Z-scores and percent identities were calculated on the DALI server.


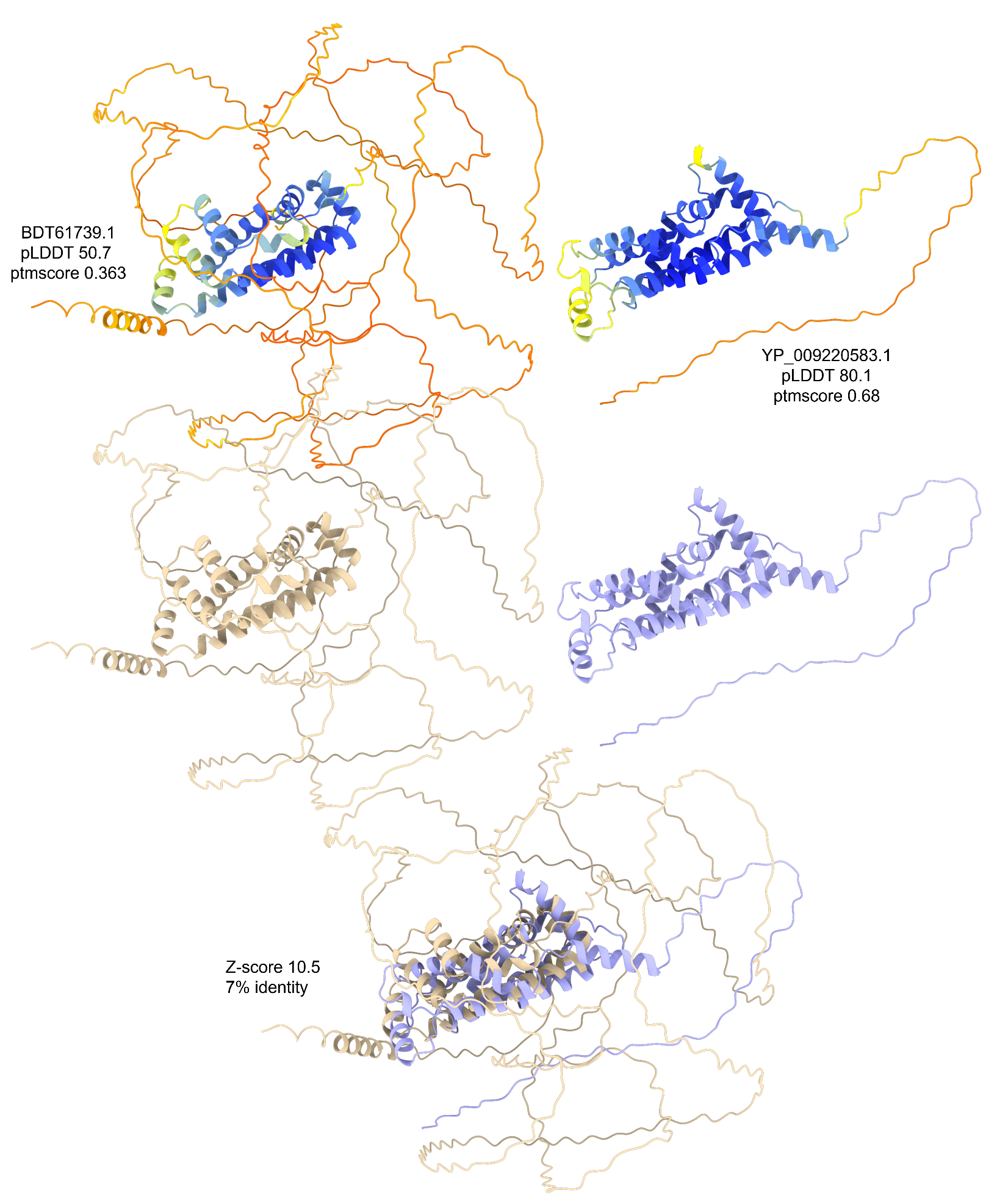


Supplementary Figure 3. Structural predictions and an alignment of wsv310 orthologs.

Top: ColabFold structural predictions of MjeNMV wsv310-like protein (left) and WSSV wsv310 protein (right). Colors indicate the pLDDT confidence measures assigned by ColabFold.

Middle: The same structural predictions as the top, with colors ignored.

Bottom: Matchmaker alignment of the predicted protein structures. Z-scores and percent identities were calculated on the DALI server.


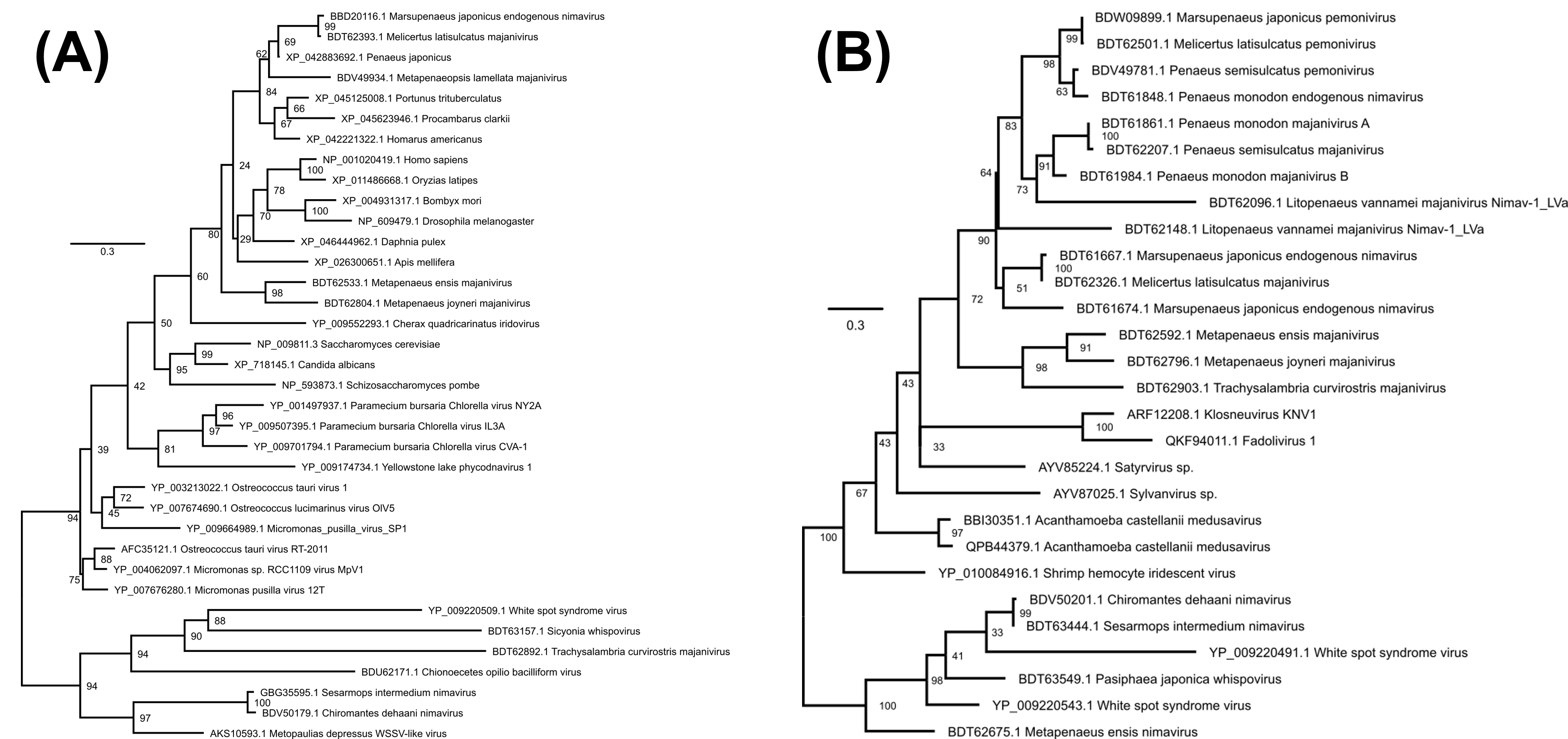


Supplementary Figure 4. Maximum phylogenetic trees of wsv112 and wsv206-like proteins

1. Maximum-likelihood phylogenetic tree of wsv112 (dUTPase)-like proteins (112 sites; model: LG+G4).
2. Maximum-likelihood phylogenetic tree of wsv206-like proteins (111 sites; model: LG+G4).


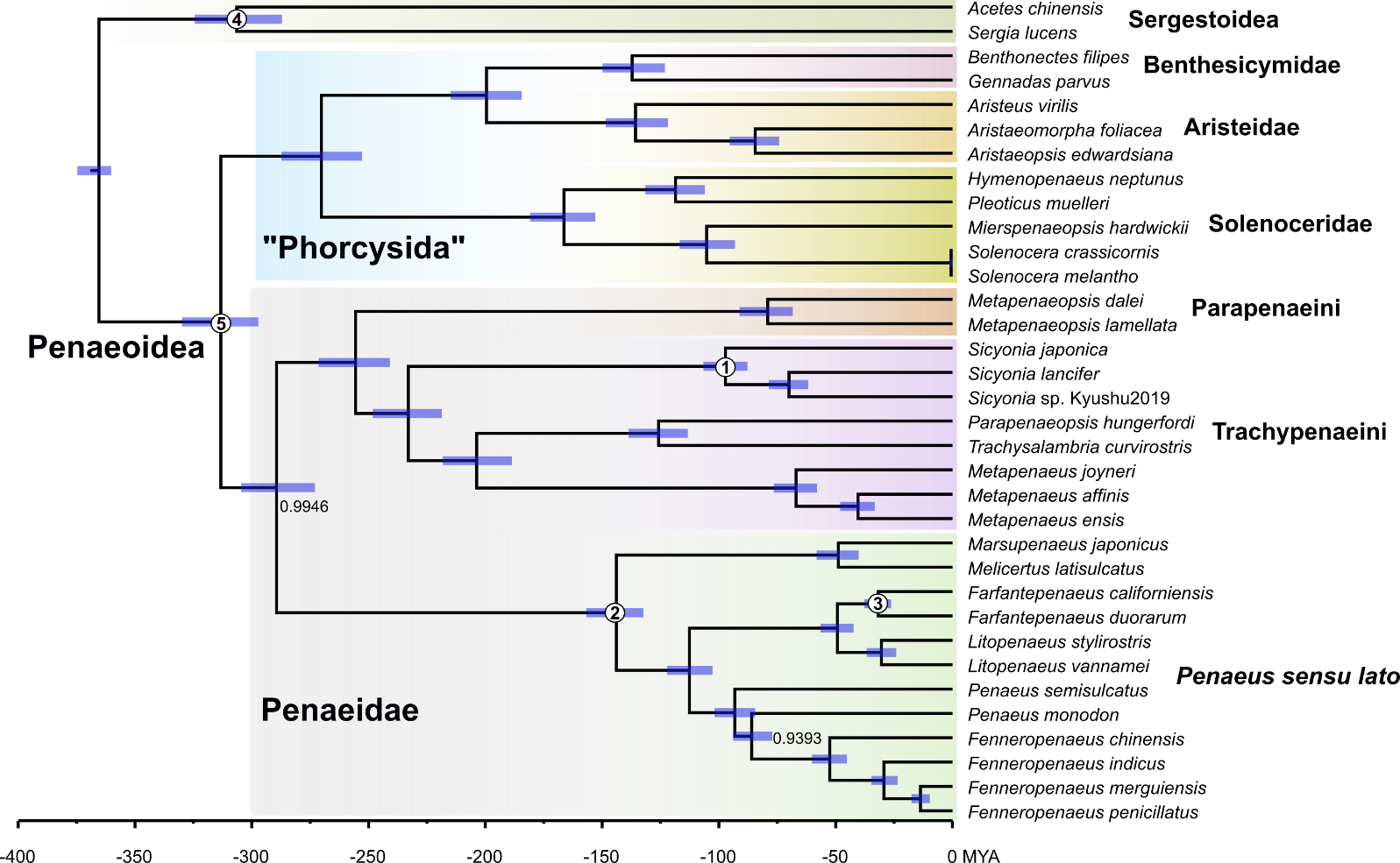


Supplementary Figure 5. Divergence time estimation of Dendrobranchiata.

A total of 13 mitochoondrial protein sequences (3,776 sites) were used in the analysis. Blue bars indicate 95% confidence intervals of estimated divergence dates. Circled numbers on nodes correspond to calibration points described in Supplementary Table 5. Posterior probabilities for the nodes were 1 unless indicated beside the corresponding nodes. Family Penaeidae was recovered as a monophyletic clade, but the relationships between the subclades (Parapenaeini, Trachypenaeini, and Penaeini, composed of *Penaeus sensu lato* in this tree) were different from previous studies. Sicyonidae, including *Sicyonia japonica*, *S. lancifer*, and *Sicyonia* sp. Kyushu2019, was nested within Trachypenaeini.


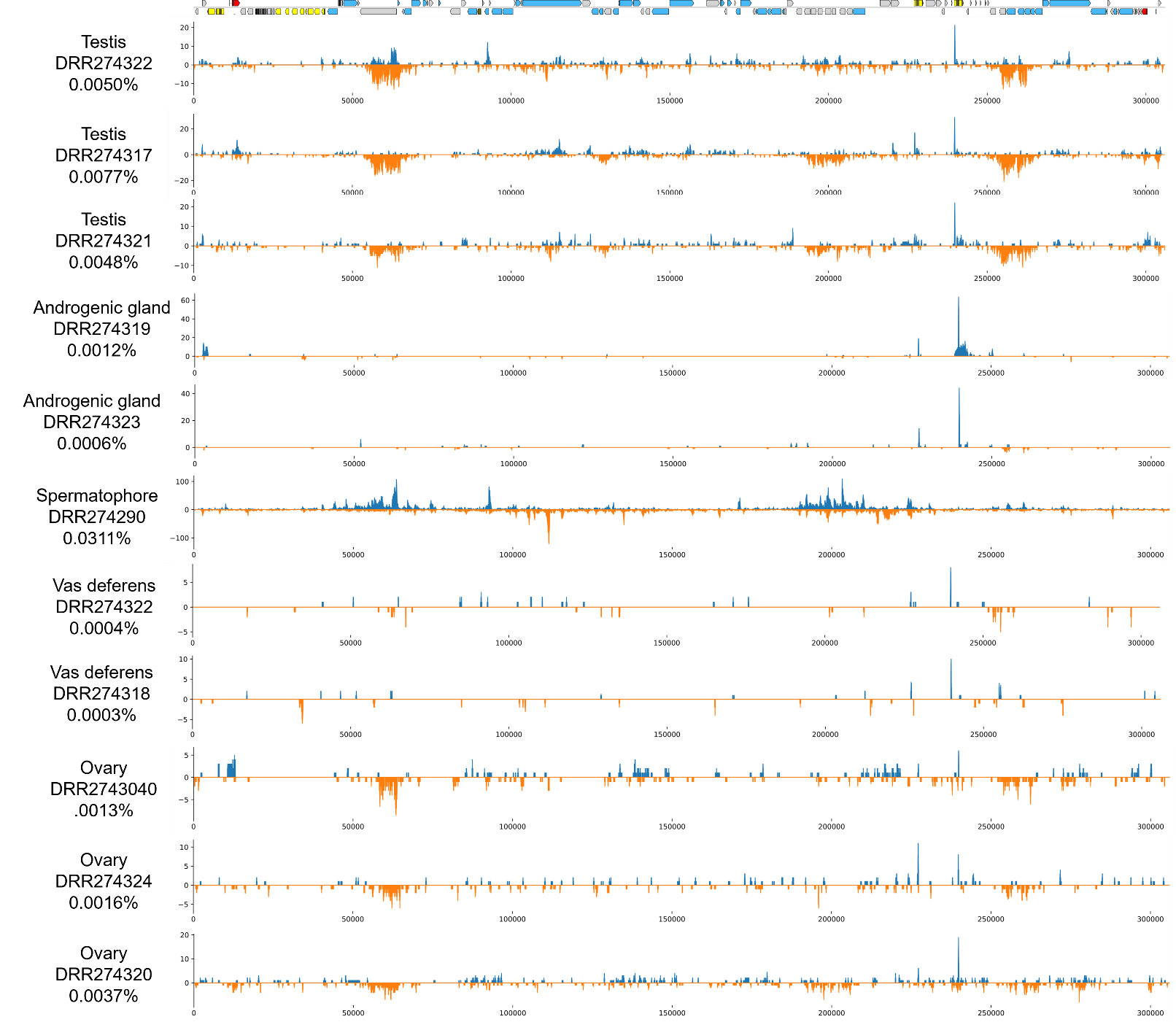


Supplementary Figure 6. RNA-seq reads mapped onto the MjeNMV genome.

The MjeNMV genome diagram is shown on the top, followed by diagrams showing RNA-seq read mapping depths derived from each library. Transcripts of left-to-right orientation are shown in blue, whereas those of right-to-left orientation are shown in orange. The left column indicates the tissue, NCBI DRA accession number, and the mapping rate (100×number of mapped reads/total number of trimmed reads).


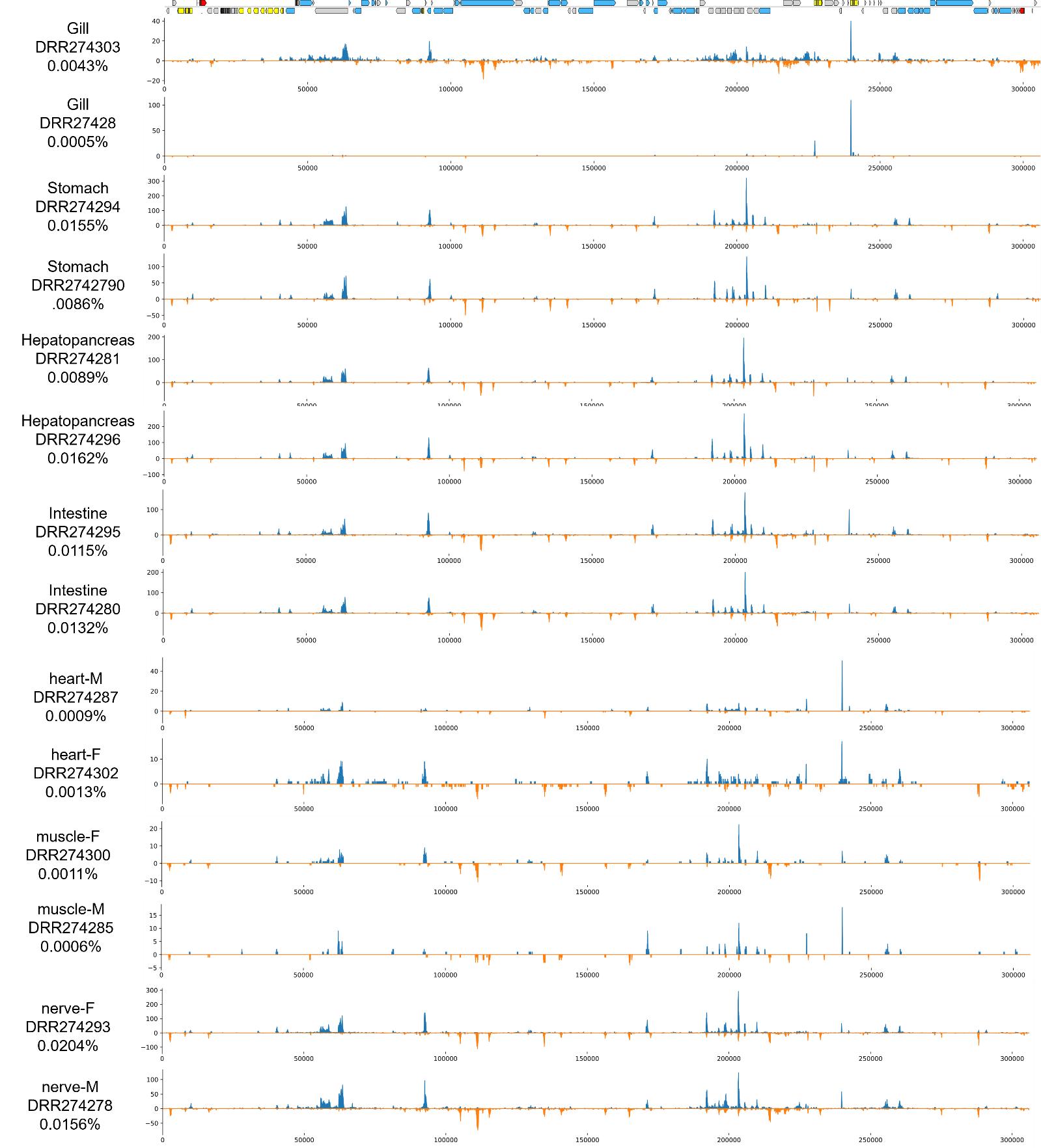


Supplementary Figure 6. RNA-seq reads mapped onto the MjeNMV genome (continued).


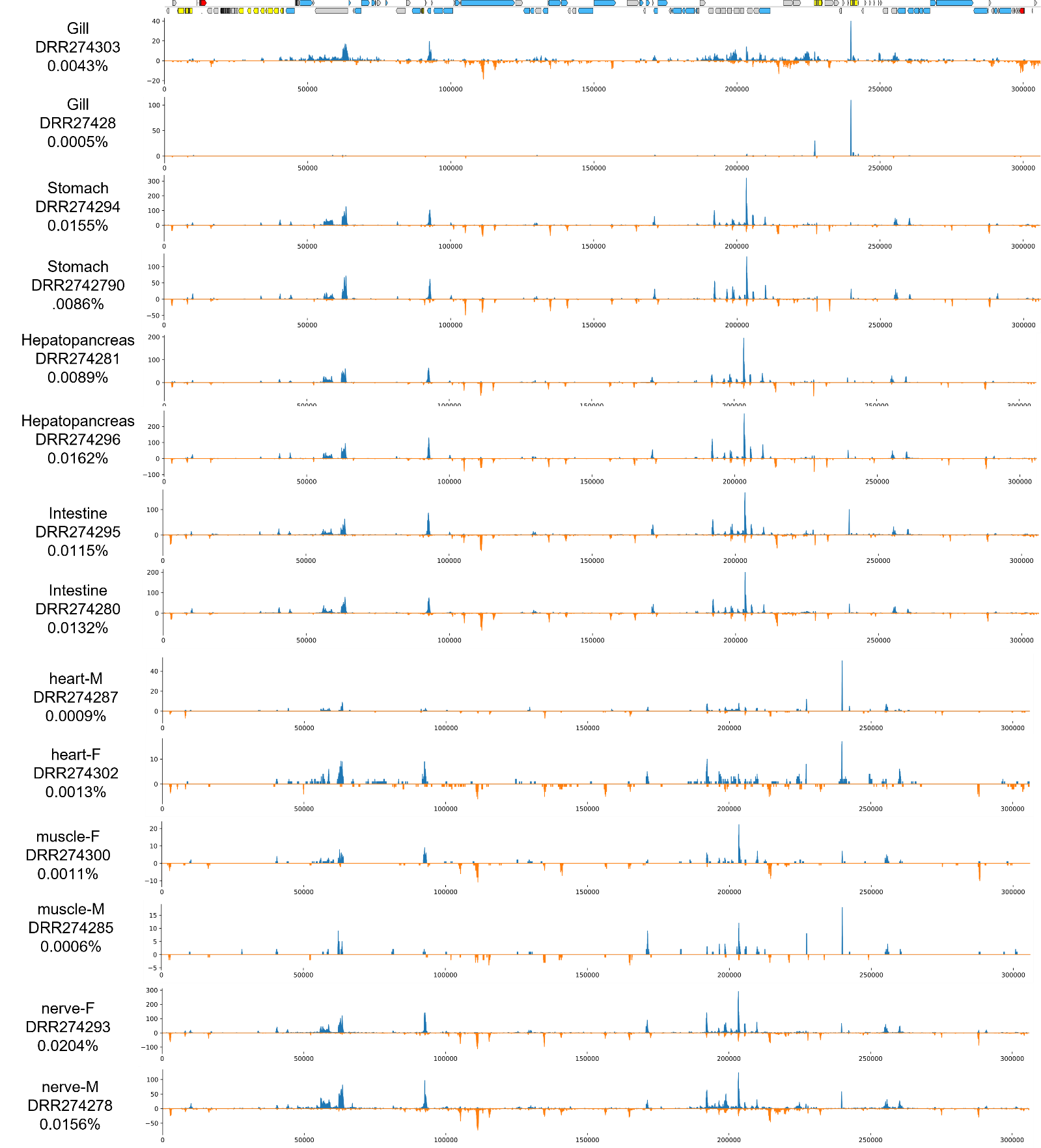


Supplementary Figure 6. RNA-seq reads mapped onto the MjeNMV genome (continued).


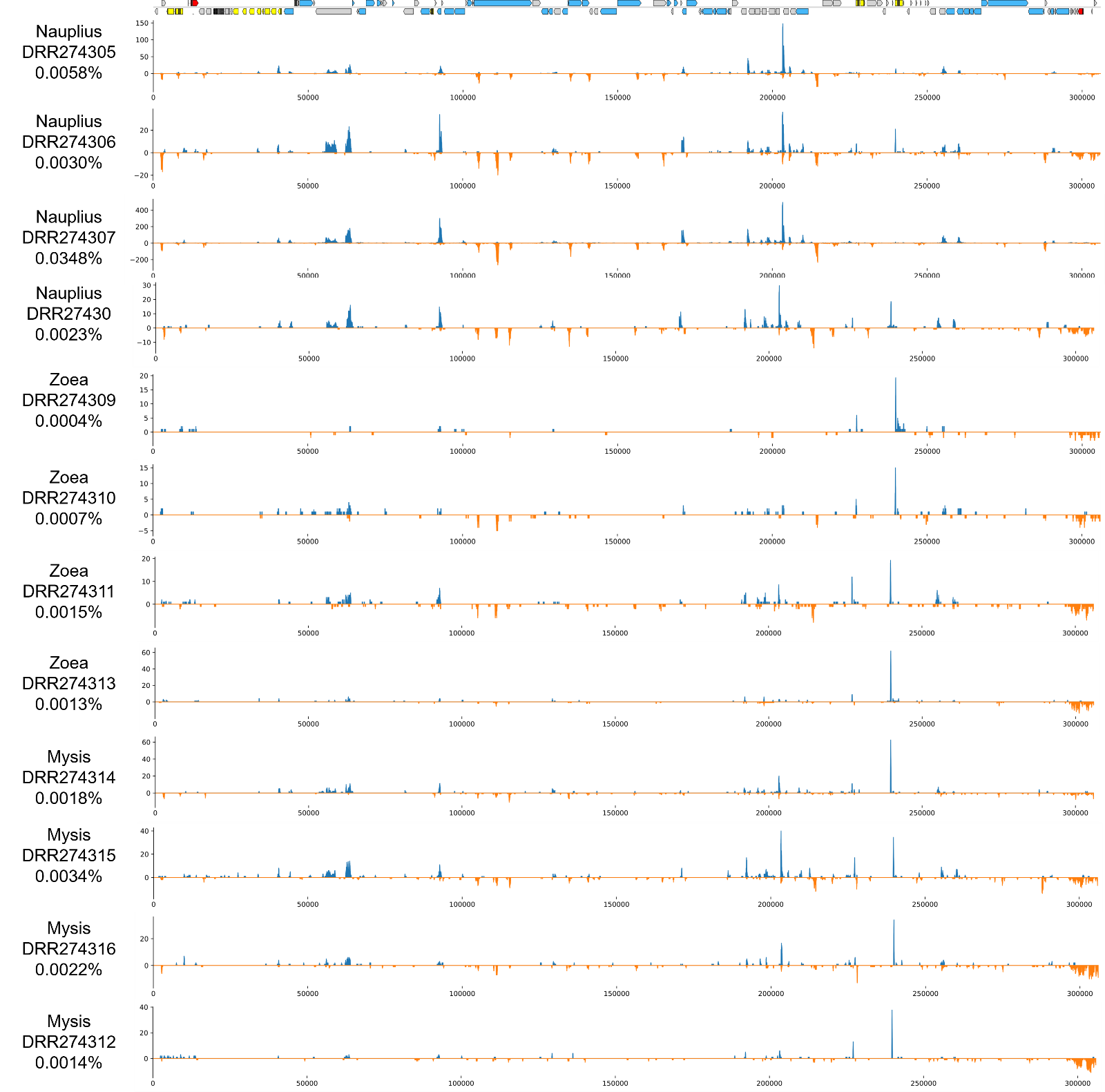


Supplementary Figure 6. RNA-seq reads mapped onto the MjeNMV genome (continued).
